# Challenges encountered in comparing international policy responses to COVID-19 and their effects

**DOI:** 10.1186/s12961-021-00783-1

**Published:** 2021-10-30

**Authors:** Christine Riley, Bo Xie, Anjum Khurshid

**Affiliations:** 1 School of Nursing, The University of Texas at Austin, 1710 Red River St., Austin, TX 78712 USA; 2 School of Information, The University of Texas at Austin, 1616 Guadalupe St, Suite #5.202, Austin, TX 78701 USA; 3 Dell Medical School, The University of Texas at Austin, 1501 Red River St, Austin, TX 78712 USA; 4 Austin, Texas USA

**Keywords:** Coronavirus, COVID-19, Public policy, Health policy

## Abstract

**Background:**

A variety of policies have been implemented around the world in response to the COVID-19 pandemic. This study originally aimed to identify and compare policy responses of different countries and their effects on the pandemic. It quickly evolved into an identification of the heterogeneity among existing policies and the challenges in making meaningful comparisons of the impact of these policies.

**Methods:**

The process of collecting and comparing data from different sources was analysed through inductive thematic analysis to understand the obstacles that impede research designed to compare COVID-19 data and related policies.

**Results:**

We identified the following obstacles: (1) no single reputable source of information and too much noise; (2) a lack of standards for how to measure and report data across countries; (3) variations in the content, implementation and enforcement of policies; and (4) politics, instead of science, leading the efforts in pandemic management.

**Conclusion:**

Heterogeneity in existing policies makes it challenging to compare the effects of various policies worldwide on the COVID-19 pandemic. Our findings call for an automatically updated informatics infrastructure across the globe for collecting and maintaining standardized data from multiple sources. There is a strong need for steadfast utilization of scientific and technical experts to inform and influence health policy. Increased investment in public health and emergency planning is essential to overcome the current pandemic, as well as future public health emergencies. Focused leadership and collaboration from world leaders in a unified mission to decrease the mortality and morbidity of the COVID-19 pandemic is imperative.

## Background

The COVID-19 pandemic is not only a public health crisis but also a major policy crisis. How different countries have reacted to the pandemic has created an ongoing natural experiment in how policy and politics affect the trajectory of a public health crisis. Some countries acted quickly to address the pandemic, but others waited. Some have relied on technocrats; others, on popular sentiment. Some have been led by science, others have followed political ideology, and some have considered implications for the future whereas others simply reacted to the immediate moment [1, 2]

Many countries have had vigorous debates about what governments should do, whether or not lockdowns should take place, to what extent privacy should be protected, and how to balance the trade-off between health and the economy [2]. More research is needed to compare the effects of existing policies and improve decision-making. Identifying obstacles that impede data-driven decisions is highly relevant to future actions taken by states and countries to manage the current and future pandemics.

### Existing efforts and limitations

Efforts to collect timely, accurate and complete data about confirmed cases, mortality, testing rates and contact tracing for COVID-19 began quickly. The Johns Hopkins Coronavirus Resource Center (JHU CRC) is an interdisciplinary collaboration among multiple schools in the university, which collects, analyses and presents data from 188 countries. Their tracking maps include a world map and a United States map, each of which was first shared with the public on 22 January 2020, and they are updated at least daily [3].

WHO also has a live Coronavirus (COVID-19) Dashboard that displays a global overview, regional overview and options that allow one to zone in on specific countries, areas and territories, as well as to compare up to nine countries, areas or territories with an overlay feature. This dashboard is also updated at least daily [4]. The United States Centers for Disease Control and Prevention (CDC)’s COVID-19 website has been the main source for the latest coronavirus information in the United States for data collection, with guidance for the public and healthcare professionals. COVID-19 case counts, deaths and laboratory testing numbers are also updated daily [5]. Another source for United States COVID-19 data was the COVID Tracking Project, a volunteer organization launched from *The Atlantic* [6]. The University of Oxford, in conjunction with the Blavatnik School of Government, has started a Coronavirus Government Response Tracker [7], which compares the strictness of government policies with a stringency score. Although this is a useful research tool, it has limited updates, and it is not included in the data analysis of WHO, JHU CRC or Our World in Data (OWID).

These existing efforts have limitations. Even with daily data updates, conclusions are limited by the accuracy and reliability of data sources in each country. The JHU CRC uses manual and automatic updates for their global map [3], but these have different frequencies and data cut-off times. Other limitations include a lack of uniformity across available data sources and discordant inclusion and exclusion criteria for calculating the numbers of positive tests, reported cases and deaths across data sources. Thus, all data are subject to continuous verification and change—a limitation acknowledged by WHO [8].

Outside influences can also impede data collection and publication efforts. Official data from the CDC, traditionally considered to be reliable and carefully vetted by scientists and public health experts, had allegedly been undermined by political meddling for most of 2020 [9].

### The present study

This study originally aimed to identify and compare policy responses of different countries and their effects on the pandemic. It quickly evolved into an identification of the heterogeneity of existing policies and the challenges in making meaningful comparisons of the effects of these policies. In the present paper, we report on challenges encountered when trying to compare COVID-19-related policies across the globe. The findings did not come from a systematic analysis of a sample. Rather, we encountered challenges when collecting data for analysis and decided to refocus research efforts on analysing the challenges encountered in the research process.

There are many studies that detail the numerous challenges of the COVID-19 pandemic, but fewer studies highlight research-related challenges during the pandemic. Some studies have mentioned research-related challenges [2, 10, 11], but not from the perspective of a policy research study. To our knowledge, no other research team has systematically reported encountering the same set of challenges as we did while collecting data on public health policies related to COVID-19 and their associated outcomes.

Our research questions were as follows:

*Research question *1 What obstacles impede research designed to compare COVID-19 data and related policies obtained from different countries?

*Research question *2 Given the identification of such challenges to policy research on the COVID-19 pandemic, what recommendations can be made to overcome those challenges?

## Methods

Our data collection started with the JHU CRC world map webpage, which was used on 1 June 2020 to select the top 20 countries with the most confirmed cases of COVID-19. These 20 countries (in alphabetical order) were Belgium, Brazil, Canada, Chile, China, France, Germany, India, Islamic Republic of Iran, Italy, Mexico, Pakistan, Peru, Qatar, Russian Federation, Saudi Arabia, Spain, Turkey, the United Kingdom, and the United States [12].

The researchers then determined which data sources to use for COVID-19-related data. WHO, CDC, OWID, JHU CRC and the COVID Tracking Project each have their own webpage and COVID-19 data dashboard, but these sources did not always concur. Therefore, we decided to only use OWID to collect data from each of the 20 countries, including the date of the first reported case of COVID-19, total confirmed cases of COVID-19, deaths from COVID-19, the total number of people tested in each country and the first testing policy for each country. While pieces of this information were also available from WHO and local government sources, OWID was used for the sake of consistency, because this source had the most data available for each data point and for each of the chosen countries. OWID has been a reputable data platform, with the goal of making knowledge on “big world problems” accessible and understandable, since 2011 [13]. OWID uses data from the JHU CRC, and collects testing data from official sources such as local governments, ministries of health and centres for disease control [14]. However, the testing data available were sporadic. Most of the countries researched did not have testing data available until mid-March, and the data were submitted at varying frequencies (daily, weekly, biweekly) or at no established frequency [15].

The time and effort it took to sort through these various sources and determine OWID to be the main source of COVID-19 data for this research presented the first challenge for this team in researching COVID-19 policies. While reviewing the information on these dashboards, other impediments ensued, as there were numerous instances of different definitions, criteria and reporting standards used for COVID-19 data among these prominent sources.

The Kaiser Family Foundation (KFF) developed a global tracker of government actions, with regular updates, to track countries’ first policy responses to COVID-19 [16]. Dates and policies regarding lockdowns and social distancing were found for the majority of countries using this tool. This source was used because the KFF is known as a nonpartisan source of facts, analysis and news for policy-makers, researchers, media sources and the public [17]. However, the KFF tracker did not provide detailed information on the development, implementation and enforcement of specific policies.

Scholarly journal articles from Google Scholar were used to further research policies on masks and lockdowns because we identified no single source for this information from every country. Keywords used to search in Google Scholar included (COVID-19 OR coronavirus) AND (lockdown OR masks), AND (policy OR policies). Google Scholar was also used to identify COVID-19-related policy and challenges associated with the act of researching the COVID-19 pandemic itself, as well as responses to the pandemic from different countries. Keywords used in this search included (COVID-19 OR coronavirus) AND (health policy OR public policy) AND (research challenges OR policy challenges) AND (politicization OR politics) AND pandemic. Sources retrieved from Google Scholar included articles from a diverse set of journals: *The Lancet*, *Nature*, *JAMA*, *Environmental Microbiology*, *Clinical Infectious Diseases*, *Journal of Public Policy*, *Nations and Nationalism*, *Policy and Society*, *Evidence and Policy*, and *Policy Sciences*.

These sources were included because of the relevance of their articles to our research questions, their highly regarded reputation among researchers and their rigorous peer-review processes.

Using Google Scholar presented its own challenges. One must sort through a greater number articles from a variety of sources, in comparison to a specific database or set of databases. However, using Google Scholar can be helpful in finding recent and specific information, such as lockdown policies in Canada that may not be found in a conventional journal database.

The Google search engine was also used to gather more information on mask and lockdown policy from mass media. The inclusion of mass media can bring up questionable and potentially biased information. However, mass media is sometimes the only source of “breaking news” and information during a public health emergency, especially when research is still being performed and data still being collected at the beginning of a pandemic. Scientific articles with information on a specific topic, such as mask policy in a certain country, can be limited. The use of mass media in this research intended to include only those sources that many people would consider widely credible and that passed the Media Bias/Fact Check with a factual reporting rating of “high” or “very high” [18].

There were other challenges identified during data collection which made it difficult to carry out the original plan of conducting a comparative analysis of policy responses from different countries. During this process, however, we realized that the obstacles themselves formed different patterns and themes that deserved serious investigation. Thus, we used inductive thematic analysis to identify, analyse and report on these patterns found within the data [19]. Following the steps recommended by Braun and Clarke [19], this inductive thematic analysis process began with analysing the data collected, identifying key concepts and patterns, and regrouping that information into themes. This analysis involved an inductive, data-driven approach, as there was no pre-existing frame within which to codify the patterns that appeared. Rather, the themes were identified using a “bottom-up” approach, based on the data collected. Subsequently, the recurring patterns were coded and reviewed, and themes identified. These recurring themes focus on the challenges, how they impede COVID-19 data and policy research, and ultimately affect the actions we take during the course of the pandemic.

## Results

We identified four distinct challenges that hindered data collection and analysis of policy responses across different countries.

### Challenge 1: no single reputable source of information and too much noise

Various sources reported first confirmed cases and confirmed deaths of COVID-19. Variations could be seen in the same data from reputable sources. According to OWID, China already had 27 confirmed cases of COVID-19 as of 31 December 2019, whereas WHO recorded the first case of COVID-19 in China as 17 January 2020 [20, 21].

Research on COVID-19, because it is a novel pandemic, cannot build on a well-established base of studies or literature. The knowledge base, including data collection and design, is under constant growth and development. There have been thousands of publications on the topic, but some studies have lacked peer review or do not meet clinical trial standards [11]. Even with an abundance of information, scientifically sound data on COVID-19 are limited [11]. Studies have shown that interventions and responses to the pandemic faced uncertainty because of limited information or because the available information was not always scrutinized [2].

*Summary* Although international organizations such as WHO provide data, and the JHU CRC compiles data from around the world, these figures do not always match.

### Challenge 2: a lack of standards for how to measure and report data across countries

Across different countries, and even within individual countries, consistent reporting standards for testing, confirmed cases, deaths, hospitalizations and general reporting were lacking or needed revision. For example, for COVID-19 testing, some countries reported the number of people tested, whereas others reported the number of tests [15]. Some countries reported testing data on only a few random dates over the span of 5 months. Other countries reported data weekly or daily [15].

Furthermore, testing has evolved during the course of the pandemic, and testing tools now include more than one type of test to diagnose infections: reverse transcription-polymerase chain reaction (RT-PCR) tests and antigen tests, and antibody tests, which show previous COVID-19 infection. It is not always clear which tests are included in total testing figures; OWID aims to include only RT-PCR tests [15].

As the pandemic continues, it is important to consider the increased use of antigen tests in the United States, Canada and European countries [22], as well as the issue that these tests are not clearly accounted for by WHO, OWID, JHU CRC or CDC [8, 15, 23, 24]. Adding to the ambiguity in the reporting of test results, the Council of State and Territorial Epidemiologists has stated that a positive antigen test can be used only to identify a probable case of COVID-19, not a confirmed case [25]. With increased use of antigen tests, it remains unclear when and where such tests are accounted for, and furthermore, if and how they influence overall testing data.

Cases of COVID-19 are defined differently among different sources. The definition has changed over time to include only confirmed cases or both confirmed and probable cases. For example, the CDC defines a confirmed case as meeting confirmatory laboratory evidence for COVID-19. CDC case counts began to include both confirmed and probable cases in their count of total cases in April 2020. However, not all jurisdictions report probable cases to the CDC [5]. The COVID Tracking Project, which was the United States data source for the JHU CRC and, subsequently, the United States data source for OWID, includes “confirmed” and “probable” in their count of “total cases” [24]. The COVID Tracking Project concluded their COVID-19 data collection on 7 March 2021 [6]. WHO has different criteria for what is included as a confirmed case. A person with a positive nucleic acid amplification test would suffice, but if the person only has a positive result from an antigen test, they must meet additional criteria, such as having a symptom of COVID-19 or being a contact of a probable or confirmed case of COVID-19 [26].

With respect to COVID-19 deaths, according to OWID, three things must be kept in mind when one evaluates confirmed death figures. First, the actual death toll from COVID-19 is likely to be higher than the number of confirmed deaths, due to limited testing and determination of the actual cause of death. Second, the way in which COVID-19 deaths are reported varies from country to country, making it difficult to compare cumulative deaths among countries. Some countries may count only hospital deaths, whereas others include deaths outside of the hospital. Finally, there are delays in reporting, from weeks to even longer, which makes reported deaths on any given day less accurate [27]. In the United States, because some states report deaths daily, weekly or monthly, death counts could not be compared across states [28]. Table 1 shows the varying standards and criteria among different types of data reported.

**Table 1:** Data reporting inconsistencies

Data type	Inconsistencies in data reporting
COVID-19 testing [15]	Number of people tested vs number of tests Inclusion criteria for test results vs subset of test type Different types of tests used (viral testing: RT-PCR or antigen tests for current infection vs antibody tests for previous infection) Test result submission frequency/reporting times (weekly, daily, etc.)
COVID-19 cases	Different inclusion criteria for total cases of COVID-19—some sources include only confirmed cases, whereas some sources include probable and confirmed cases in their data
COVID-19 deaths [27]	Not all deaths outside of hospitals accounted for Actual death toll likely higher than number of confirmed deaths
COVID-19 reporting standards and timing	Changing reporting requirements [29, 30] Varying reporting frequencies [8, 28] Delays in reporting [28]

In addition to the difficulties in standards and guidelines for reporting, politics have disrupted reporting processes and changed reporting requirements months into the pandemic. In the United States, for example, President Trump mandated that states bypass the CDC in COVID-19 data reporting and instead report data to a new private contractor, TeleTracking. The president’s coronavirus task force defended this move as essential for better streamlining of data, stating that the CDC’s system was too slow [29].

Defined measurements and data reporting standards must be implemented for accurate data collection. Other researchers have argued for a standardized reporting system [10, 11, 30]. Gardner et al. found that such a system would be subject to numerous challenges, some of which were also encountered during our research process. The brief communication by Gardner et al. also identified inconsistencies in reporting medium, adaptability to changes over time, and privacy concerns as other issues encountered in their experience of COVID-19 data collection and reporting. However, this article only focused on open data standards [10]. Our study explores other challenges for policy research and policy analysis. Along with highlighting the need for consistent data collection and reporting standards, our study adds new insight into the politicization of the COVID-19 pandemic, and identifies variations in content, implementation and enforcement of policies.

Demands for streamlined reporting mechanisms for COVID-19 testing results have been voiced by testing sites (including large drugstore chains, pharmacies, private laboratories and healthcare providers) to the CDC, inquiring about options for centralized reporting of results instead of reporting the same information to multiple entities [30].

*Summary* Inconsistencies in data, even from reputable institutions, exist due to varying data cut-off times, different inclusion criteria and varying standards for data. These incompatible standards do not allow “seamless aggregation and processing of the data”, impede dynamic analysis and lengthen the policy development process [10].

### Challenge 3: variation in the content, implementation and enforcement of policies

The research team focused on policies intended to mitigate the spread of COVID-19, such as mask-wearing, lockdowns, social distancing and testing strategies. Variations in the implementation and enforcement of similar policies within the same country and across different countries warrant further research on the strictness of specific policies and levels of enforcement for public adherence.

For example, mask policies in Belgium required everyone over 12 years of age to wear masks on public transport [31]. Other countries such as the United States [32] and Canada [33] “advised” or “recommended” masks. Variation in mask policy was also found within single countries. Brazil initially required masks only in certain cities [34], as did Italy [35].

Similar to the many variations in policies for masks, lockdowns were implemented and enforced in different ways. For example, Italians had to carry a self-certification document stating their reason for not being in their dwelling—work, health, visiting relatives or an emergency [36]. During the initial phase of Italy’s lockdown, people were not allowed to be more than 200 yards away from home, and healthcare workers were told not to request leave [37].

Many other lockdown variations existed among countries, with only some cities, areas or neighbourhoods under lockdown, as in Chile, Saudi Arabia, Spain [16], Pakistan [38] and the United States [39]. Table 2 provides examples of variations in mask and lockdown policies for different countries.

**Table 2:** Variations in mask and lockdown policies

	Mask policies and their effective dates	Lockdown policies and their effective dates
Belgium	4/5/2020: mandatory face masks on public transport for everyone > 12 years [31]	18/3/2020: police-enforced general lockdown; essential errands only [16]
Brazil	22/4/20: face masks required in public in certain cities [34]	8/4/20: statewide quarantine [16]
Italy	6/4/20: masks compulsory when one goes outside (residents of Lombardy) [35]	8/3/2020: general lockdown across components of everyday life; businesses can operate if they implement safety measures [16]
United States	3/4/20: CDC recommends masks in public [32]	Lockdown dates varied by state; earliest lockdowns in CA on 19/3/2020, and NY 22/on 3/2020 [39]

Policy variations exist for many reasons, especially when leaders do not know what the best response to a given crisis might be. Emergencies can force leaders to utilize limited data and research or “evidence-enough” when quick action is needed [40]. There are “almost limitless means of combining [policy tools], as well as varying degrees to which tools can be applied (intensity) and sequenced” [2, 41, 42]. Durations of policies also vary. Researchers and policy analysts must consider governments’ non-decisions or inactions, which can be just as important as concrete actions [43].

*Summary* Policy variations among similar actions, such as mask-wearing and lockdowns, make it difficult to compare the impact of specific policies within single countries and among countries. Understanding the differences in policy responses can determine where improvements are needed for the current crisis, as well as future crises [2].

This type of policy analysis can aid planning and public health security, possibly enabling us to avoid more frequent lockdowns of longer durations and subsequently curtail the economic impact of the virus, already seen in bankruptcies, job loss, decreasing tax revenues and other economic costs [44].

### Challenge 4: politics, instead of science, leading the efforts in pandemic management

Data collected from countries with strong censorship, where governments and political leaders themselves control media sources and other public information, often warrant further scrutiny to ensure the data’s accuracy and reliability. The following are examples of pandemic politicization that disrupt data collection and analysis and interfere with ongoing research.

After COVID-19 had already been declared a pandemic by WHO, President Trump minimized the severity of the public health emergency on 8 May 2020, stating that “This is going to go away without a vaccine” [45]. Later, on 6 July 2020, President Trump announced that the United States would officially withdraw from WHO and redirect funds to other United States global health priorities. This move came with much controversy and dismay from academic and political leaders in the United States and around the world [46].

Further evidence of potential political interference was seen when the CDC published guidance suggesting that people who might have been exposed to COVID-19 did not necessarily need testing, a recommendation that it reversed the following month, as well as guidance on airborne spread of the coronavirus, which was withdrawn in 2 days. The CDC stated that the guidance was published in error [47].

Like President Trump, President Jair Bolsonaro of Brazil took a political approach to the pandemic that involved denial and impaired the country’s ability to efficiently manage the crisis. Both leaders repeatedly resisted recommendations made by scientific experts, as illustrated by President Trump’s cavalier attitude towards masks throughout the beginning of the pandemic [48], followed by his abrupt reversal and final endorsement of masks in mid-July. Bolsonaro opposed physical distancing [1]. These actions downplayed the seriousness of the pandemic and led many people in the two countries to question official guidance.

Even though the health institutions of individual states in the United States and Brazil have developed their own policies in response to the pandemic, the approaches of both nations’ leaders have put their large countries at risk and have made it more difficult for local leaders to develop effective policy. Federal leadership has focused largely on economics and political outcomes (e.g., re-election for Trump in 2020 and for Bolsonaro in 2022).

President Xi Jinping of China has also been criticized for his handling of the pandemic. Xi has been accused of inaction in early January; critics have indicated that he knew about a novel virus and its potential to spread, but that local leaders were not authorized to disclose information about the growing outbreak without the president’s approval. Suppression of civil society has been seen in China before [49], and there were attempts to suppress information from the scientific community about the outbreak, as in the case of Dr Li Wenliang, who died from the virus after he was detained and disciplined by authorities for trying to warn his colleagues during the early stage of the outbreak in Wuhan [50].

The COVID-19 pandemic has highlighted not only the importance of effective leadership in individual countries, but also the need for global collaboration and sharing of information and resources among nations’ leaders. As the global population increases, we increasingly depend on international interactions. At the same time, as populations grow and spread, we increasingly share our spaces with different animals, and new viruses emerge in the human population.

*Summary* With self-serving leadership, misinformation is bound to spread, causing unnecessary levels of uncertainty and stress among the public [51]. The internal conflicts in Brazil and the United States among local leaders, scientific experts and presidents have sparked public controversies and “blame games” [43]. An objective, collaborative, sustained approach to the pandemic from national and state leaders was and still is needed. Many conflicts in decision-making may be avoided if there is a push for evidence-based policy-making. If health policies and government actions are transparent and clearly justified to the public, there is more likelihood for approval, trust and buy-in from the public [43, 51].

## Discussion

Each of the challenges presented above had unique implications, which helped to suggest recommendations and potential solutions to address the issues contributing to each challenge.

### Recommendations to address challenge 1

With COVID-19 data sources reporting similar but not identical information on the pandemic, the information provided can be counterproductive and inefficient. Researchers conducting data analysis, public health departments, epidemiologists and the public need a reliable source of information. Inconclusive data and premature analyses may lead to misinformation, which can divert limited resources from testing and therapeutic development and can thwart policy efforts. This time-consuming research can also delay the policy process as policy-makers endeavour to ensure that research findings are accurate and up to date.

The development of a reliable, automated public health informatics infrastructure to collect massive amounts of data in real time, along with the uptake and utilization of such an infrastructure among countries, is a long-term recommendation for synthesizing and standardizing data collection and analysis. A continuously updated infrastructure could be an efficient tool for determining critical responses to an ongoing pandemic, but will take time to develop, implement and utilize.

Current data tables and dashboards for the COVID-19 pandemic present conflicting information, but they can be useful for the creation and shaping of an international informatics infrastructure, which could be applicable not only for pandemics, but also for tracking, identifying and monitoring other public health emergencies. Such a reliable data-tracking system could facilitate the development and improvement of preparedness plans, enabling all countries to look at what has been tried and tested in other parts of the world and what might work in specific areas. The development of an automated data collection and sharing system could be initiated and accelerated as more public institutions, research agencies and health departments begin to see where they can currently synchronize and streamline information.

Researchers are making a case for automated data collection and data sharing, with a “standardized reporting system for systematically collecting, visualizing and sharing high quality data on emerging infectious and notifiable diseases in real-time” [10, 11]. A critical component of such a system would be the democratization of data, with all collected information based on open data standards, yet maintaining necessary privacy controls [10].

### Recommendations to address challenge 2

Defined measures must be used to report data, with agreements about terms and standard definitions to simplify comparisons among countries. Definitions should include, but are not limited to, social distancing, face masks, lockdowns, stay-at-home orders, essential errands, essential workers, criteria for COVID-19 testing data to be reported, and criteria for COVID-19 deaths to be reported. The development of definitions and reporting standards is a short-term recommendation.

This recommendation could be implemented if more news sources, scientific journals, data collection entities, public health agencies, universities and other research institutions discussed inclusion and exclusion criteria for commonly used terms and agreed upon standards. These standards and definitions would not dictate that the country has to have the same definition for every term, but a set of standards, criteria and definitions should be communicated as clearly as possible to local leaders and public officials, and understood by the public and outside leaders.

For example, there are many considerations regarding the types of tests reported in testing for COVID-19. It is not helpful to compare total testing data from two countries if one country includes antigen tests in their testing figures but another country that uses antigen tests does not include those tests in its testing results. Data and policy responses of specific countries can be more manageable and easily comparable if countries agree on defined standards.

Lower- and middle-income countries need additional support to respond efficiently to COVID-19 [52]. Standard definitions, as well as a global informatics infrastructure, would be especially beneficial for developing countries and those with healthcare systems that are not as robust as those of more developed countries.

### Recommendations to address challenge 3

Policy trackers, such as those built by the KFF and the Coronavirus Government Response Tracker, can be built upon. Additionally, there is a need for increased preparedness, readiness and response actions for public health emergencies [51, 52].

Governments should increase investments in infrastructure for preparedness, readiness and response to public health emergencies. World leaders must first agree with public health officials that investing in public health infrastructure is a priority investment in our future, which will save valuable time and lives. As soon as possible, national and federal governments should make all efforts to allocate increased funding towards public health departments, infection control and prevention efforts, emergency response planning, and a secure a robust healthcare system infrastructure. Contingency planning is costly, but it avoids future costs associated with a more reactive approach to any health crisis for which a nation is ill-prepared.

A proactive approach for public health emergencies such as the coronavirus pandemic must include significant budgetary commitment and investment in our public health and emergency planning programmes. This will not be the last major health crisis. Preparation and prevention are key and will enable us to act quickly and contain the spread of harmful pathogens.

We can learn from past epidemics, saving valuable time and lives, if we collaborate in research, including information sharing. Vietnam, South Korea and Singapore have all experienced previous outbreaks of Middle East respiratory syndrome (MERS) or severe acute respiratory syndrome (SARS) epidemics in the past and have displayed remarkably effective responses to COVID-19, with better control of viral spread and significantly fewer confirmed cases and deaths attributed to COVID-19 [53, 54]. However, every country should not have to experience an epidemic to know how to respond to such an event.

### Recommendations to address challenge 4

A pandemic is, by definition, a global crisis that necessitates an international response. World leaders should make every effort to collaborate with scientific and technical experts and join forces with other countries in defeating pandemics. Mutual goals of identifying and developing successful policies could improve response efficiency and ameliorate the negative effects of any pandemic. While some policies do require quick decision-making, it is all the more important to review policy options, especially where there may not be enough evidence to support a specific policy, with other leaders, scientists and subject matter experts.

Objectives and goals should be discussed and clearly defined as soon as possible. These objectives and goals, as well as data and other evidence for policy action, should be frequently evaluated in relation to each other, to determine whether the current course of action needs revision. With consistent, accountable, reliable leaders who can put politics aside and let science and data speak for themselves, it will be easier to focus on implementing a national or international informatics infrastructure to continuously collect and monitor data from around the world.

As political leaders increasingly incorporate science-based knowledge and judgements into public policies, the accountability of political leaders and policy-makers becomes more complicated [43]. Today, to inform policies that protect the public, global leaders must confer with subject matter experts to make informed decisions, and they must ensure that reliable data are readily available for accountability.

*Summary* There are specific recommendations to address each of the four research-related challenges identified in this manuscript. Table 3 provides a summary of these recommendations and their associated action items.

**Table 3:** Summary of recommendations and action items

Recommendations	Action items
A comprehensive, automated international public health informatics infrastructure is needed to enable fast, accurate and complete data collection	Identify reliable sources of up-to-date and easily accessible information Engage research communities, health departments and public health agencies in discussion of what is needed for comprehensive data collection and plans to establish data collection tools and infrastructure
Define and agree upon standards among countries regarding data elements for testing, death counts and confirmed cases that are essential for data collection and reporting. Definitions for social/physical distancing, masks (face mask, cloth face covering, surgical mask, N95 respirator), lockdowns, “stay-at-home” orders, essential errands and essential workers must be standardized	Engage research communities, health departments and public health agencies in collaboration towards development of standards and definitions for commonly used terms related to this and future pandemics
Investment in public health and emergency preparedness must increase. The robustness of existing healthcare systems must be improved to ensure that citizens have fair and equal access to healthcare and health maintenance resources	Scientific communities must be empowered to assess and scrutinize government policies, actions and inactions. These entities must use an evidence-based approach to identify and demand allocation of more resources and investment where they are needed Subject national health systems and emergency plans to international benchmarks [51]
Scientific and technical experts must be included in developing evidence-based policy with public buy-in and accountable leadership	Public health agencies, local governments and federal governments must incorporate scientific and technical expertise in task forces that gather and assess data and establish policies based on evidence. Scientific expertise, collaboration and explanation can help engage the public in all aspects of crisis preparedness Share contingency plans with the public and offer options for public comment

### Target audience for implementing recommendations

The target audience for these recommendations comprises national and federal government officials, including local and state health departments, the agencies for disease prevention and control in each country, and WHO. The institutions responsible for protecting public health must implement policies to achieve adequate protection.

Political leaders, administrative directors, emergency managers, epidemiologists, policy analysts and infectious disease specialists all have roles in crisis response management at strategic and operational levels to provide information to the public and to support coordination and collaboration [43].

These recommendations also apply to universities that conduct research, especially in public health, public policy, informatics, nursing and medicine. Scientific and professional organizations must work to inform and influence local and national governments about their research and provide their expert knowledge proactively.

The healthcare industry will undoubtedly benefit from reliable and accurate data, because it supplies the frontline healthcare workers who immediately respond to confirmed cases of viruses such as COVID-19. Accurate data and informed public health policies are critical to the healthcare industry, to prevent essential workers from becoming burdened and overwhelmed during pandemics.

Other industries and the general public can also benefit from a reliable data infrastructure, because pandemics affect the global economy. Industries such as the manufacturers of personal protective equipment need reliable estimates to determine production. People’s livelihoods have been devastated by loss of work, business and income. Reliable data and efficient data collection can help to ensure that effective policies are implemented, with positive “trickle-down” effects for personal and community health.

Global responses to the pandemic have escalated the need for renewed research not only on the surge in new policy decisions but also on the effects of indecision and policy termination [43]. Future research might entail a framework for categorizing the various policy tools used by countries, the development of policy compliance and levels of implementation, and the categorization of strength of evidence of policy effectiveness for pandemic outcomes.

## Limitations

Conducting research during a pandemic comes with its own challenges of managing ongoing, continuously growing information on such a rapidly evolving topic. Data are constantly being collected from numerous sources and include frequent changes and updates to previously reported data. At the same time, research on COVID-19 has been conducted at a remarkable pace, often with accelerated publication, but not always standardized review [55].

We may have missed some information during this research process because this research team did not intend to conduct a systematic literature review. Rather, the research changed directions from the original plan, and an unsystematic approach was used to gather applicable information from a variety of sources on this quickly developing topic. For future research, a systematic review and a narrower focus on a specific COVID-19-related policy and a specific COVID-19-related outcome would be helpful in conducting policy analysis.

## Conclusion

Along with sickness and death, the effects of the COVID-19 pandemic seem to leave nothing untouched. Existing problems have been exacerbated. Fragile economies in developed and developing countries have contracted amid lockdowns and market fluctuations. Unemployment has increased, and internal and external conflicts have intensified [44].

This paper offers a look at the beginning of the coronavirus pandemic, shows the myriad responses and efforts taken all over the world, and shines a light on the challenges encountered when conducting policy research based on rapidly evolving information during an ongoing pandemic. The challenges identified in this research have led to recommendations for improving data collection methods and mitigation strategies in the ongoing pandemic. While calling for standardization in research is not new, the identification of standards in research is especially critical in public health emergencies like a pandemic.

These recommendations may aid future policy research that aims to understand and manage public health crises. There are lessons to be learned from life-and-death decisions made during the beginning of the COVID-19 pandemic, so that we can improve our policy-making and policy analysis infrastructure. Only collaboration and coordination among countries and states and among experts across the spectrum can ensure a better global response to such crises in the future.

## Data Availability

The datasets analysed during the initial study are available in the Johns Hopkins University and Medicine Coronavirus Resource Center repository, https://coronavirus.jhu.edu/map.html, and Our World in Data repository, https://ourworldindata.org/coronavirus.
